# Effector-independent reduction in choice reaction time following bi-hemispheric transcranial direct current stimulation over motor cortex

**DOI:** 10.1371/journal.pone.0172714

**Published:** 2017-03-06

**Authors:** Neil M. Drummond, Gabrielle Hayduk-Costa, Alexandra Leguerrier, Anthony N. Carlsen

**Affiliations:** School of Human Kinetics, Faculty of Health Sciences, University of Ottawa, Ottawa, Ontario, Canada; University Medical Center Goettingen, GERMANY

## Abstract

Increased reaction times (RT) during choice-RT tasks stem from a requirement for additional processing as well as reduced motor-specific preparatory activation. Transcranial direct current stimulation (tDCS) can modulate primary motor cortex excitability, increasing (anodal stimulation) or decreasing (cathodal stimulation) excitability in underlying cortical tissue. The present study investigated whether lateralized differences in choice-RT would result from the concurrent modulation of left and right motor cortices using bi-hemispheric tDCS. Participants completed a choice-RT task requiring either a left or right wrist extension. In forced-choice trials an illuminated target indicated the required response, whereas in free-choice trials participants freely selected either response upon illumination of a central fixation. Following a pre-test trial block, offline bi-hemispheric tDCS (1 mA) was applied over the left and right motor cortices for 10 minutes, which was followed by a post-tDCS block of RT trials. Twelve participants completed three experimental sessions, two with real tDCS (anode right, anode left), as well as a sham tDCS session. Post-tDCS results showed faster RTs for both right and left responses irrespective of tDCS polarity during forced-choice trials, while sham tDCS had no effect. In contrast, no stimulation-related RT or response selection differences were observed in free-choice trials. The present study shows evidence of an effector-independent speeding of response initiation in a forced-choice RT task following bi-hemispheric tDCS and yields novel information regarding the functional effect of bi-hemispheric tDCS.

## Introduction

Choice reaction time (RT) tasks require participants to react to one of multiple possible stimuli with corresponding responses. The time to initiate the appropriate response is typically found to be longer than in a simple RT task, in which a single required response is known in advance [1]. These RT differences have been suggested to result from the additional processing required to select and execute the proper response in a choice-RT task [2]. Although some motor readiness is observed when the required response is not known in advance [3], most studies have shown that motor preparatory activation related to the response is limited in choice-RT tasks. For example, electroencephalography (EEG) studies have shown that the negative-going shift in electrical potential observed during the preparatory period (known as the readiness potential) is smaller for choice compared to simple RT tasks. Specifically, the amplitude of the readiness potential is thought to reflect the amount response-specific motor preparatory activation [4, 5], and there is a reduction in the amplitude of the motor-related readiness potential with increased uncertainty about the upcoming response [6]. Indeed, neural accumulator models predict that the closer the activation level of the motor output is to the fixed threshold needed to initiate a response, the faster it should be initiated [7]. Since limited motor preparation can occur during choice-RT tasks, the observed increased RTs may be attributable in part to lowered baseline activation.

One method used to modulate cortical activation is transcranial direct current stimulation (tDCS). By applying a weak electrical current over the scalp, polarity-dependent changes have been shown whereby anodal stimulation increases, and cathodal stimulation decreases the excitability of underlying neural tissue (see [8] for a review). These excitability changes can in turn influence observed behavior, including but not limited to increased performance of a serial RT task [9], early response initiation in an anticipation timing task [10], and speeded simple RT [11]. In the current study it was reasoned that by using a bi-hemispheric tDCS montage, anodal tDCS could be applied over one hemisphere while concurrently applying cathodal tDCS over the other, leading to up-regulation of excitability in one hemisphere and down-regulation in the other [12, 13]. It was hypothesized that if the slower RTs observed in choice-RT tasks are related to differences in cortical excitability, RTs may be modulated following tDCS resulting in faster RTs in the limb contralateral to the hemisphere receiving anodal stimulation and slower RTs in the limb contralateral to the cathodal stimulation. It was also hypothesized that an increased proportion of responses would be made with the limb contralateral to the cortical hemisphere receiving anodal (excitatory) tDCS during free-choice trials.

## Materials & methods

### Participants

Twelve neurologically healthy volunteers (6M; 27.7 years, SD = 11.2) who self-reported as right handed participated in three experimental sessions on three separate days involving real tDCS applied in two sessions and sham tDCS applied in one session. A power calculation using GPower 3.1.9 was used to determine the sample size required to detect an effect size (.556) similar to that previously reported for the effect of tDCS on a simple RT task [11]. Although this previous study investigated the effect of tDCS applied over SMA rather than M1 as used here, other parameters of the stimulation and testing protocol were more similar than those of other studies. The power calculation was performed for an F-test involving repeated measures, within-factors effects (alpha = .05, power = .8, correlation among repeated measures = .5) indicating that a sample size of 9 would be adequate. Written informed consent was obtained before beginning testing, and the study was approved by University of Ottawa Research Ethics Board (REB approval: H03-12-03), and conducted in accordance with the latest revision of the Declaration of Helsinki.

### Apparatus

Participants sat facing a computer monitor at eye level, approximately 1 m away. Both forearms were placed in custom manipulanda which allowed flexion/extension of the wrists in the transverse plane. Both hands were semi-pronated with the palms facing inward in a neutral position (neither flexed nor extended), and secured to a swivelling rest with the axis of rotation at the wrist. The shoulders were flexed and abducted approximately 15° with the arms secured in armrests using two Velcro straps located between the wrist and elbow.

### Task

Participants performed a choice-RT task requiring a 20° wrist extension with either the right or left wrist upon illumination of an associated stimulus. A black central fixation circle (1 cm diameter) was displayed on the computer screen, along with 3 x 3 cm black squares located 4.5 cm to the right and left of fixation. At the beginning of each trial, a tone (100 ms, 200 Hz, 82 dB) sounded and the words “Get Ready!” were presented for 2 s above the central fixation. This was followed by a 1500–2000 ms random foreperiod prior to the illumination of the imperative stimulus (IS), which involved one of the boxes turning bright green. Participants were instructed to initiate a right wrist extension upon illumination of the right box, left wrist extension upon illumination of the left box, and if the central fixation circle illuminated they were instructed they had free-choice of either movement (right or left extension). Prior to testing, participants were informed that during free-choice trials, it did not matter which response side was chosen, only to respond with one as quickly as possible. Instructions emphasized fast reactions on all trials and a points scheme was used to encourage fast RTs. After each trial, feedback was displayed for 3 s including RT and running total of points awarded which were scaled to individual RT performance. Participants were notified if their movement amplitude error was greater than 10° and were also verbally encouraged to react as fast and accurately as possible in response to the IS.

On each testing day participants first performed 16 practice trials (7 forced-right, 8 forced-left, 1 free-choice) which were followed by a pre-tDCS testing block consisting of 100 choice-RT trials (40 forced-left, 40 forced-right, and 20 free-choice trials). Trials were pseudo-randomized whereby free-choice trials were preceded an equal number of times by right and left forced-choice trials. Following the pre-tDCS block, tDCS (or sham tDCS) was administered, followed by an 8-minute rest interval of quiet sitting (see rationale below). A post-tDCS testing block of 100 trials was then performed. This sequence was repeated each testing day.

### Transcranial Direct Current Stimulation (tDCS)

During the active stimulation (real tDCS) sessions two self-adhesive electrodes (small sponge electrode, 1.5cc, 7.8 cm^2^, Ionto+ Inc.) were placed bilaterally on the scalp over the left and right motor representations for wrist extensors. The location of the electrodes, 4.7 cm lateral and 1.1 cm anterior to the measured location of Cz (according to the International 10–20 system), corresponded to the approximate location of the right and left extensor carpi radials longus (ECR) representations on M1 [14].

Between pre- and post-tDCS testing blocks in each of the active stimulation testing sessions, tDCS was applied with either the anodal lead attached to the electrode over the right motor cortex and the cathodal lead attached to the electrode over left motor cortex, or vice versa. Stimulation was delivered using a Dupel iontophoresis constant current delivery device (Empi Inc.). Current was set at 1 mA and was delivered for 10 minutes (current density = 0.128 mA /cm^2^). Previous research has shown that using similar stimulation parameters, tDCS effects were greatest 10–25 minutes following stimulation [11, 15]; as such, participants sat quietly for 8 minutes after tDCS completion before completing the post-tDCS block (approximately 16 min duration). Active stimulation order was counterbalanced and neither the participants nor the experimenter conducting the trials were aware of stimulation polarity. Testing days were conducted a minimum of 48 hours apart to ensure a complete washout of any residual tDCS effects.

Sham stimulation was administered with tDCS electrodes placed in a unilateral montage with one small sponge electrode (15cc, 7.8 cm2, Ionto+ Inc.) placed over either the right or left ECR motor representation and the other electrode (carbon-foam electrode, 39 cm2, Ionto+ Inc.) placed centrally on the forehead directly above the eyebrows. This different montage was used to ensure that participants would remain naïve to the purpose of the sham session, and electrode montage was not expected to have any direct bearing on the outcome. During sham stimulation, the stimulation device was only powered on while ramping up to 1 mA (<20 sec), then immediately shut off without the participant’s awareness. In six participants sham stimulation occurred during the first session, for the remaining six participants sham stimulation occurred in a third session. Sham stimulation side (right or left M1) was balanced between participants.

### Recording equipment

Surface electromyographic (EMG) data were collected from the muscle bellies of the left and right ECR and the left and right flexor carpi radialis (FCR) using bipolar preamplified (gain = 10) surface electrodes (Delsys Bagnoli DE-2.1) connected via shielded cabling to an external amplifier (Delsys Bagnoli-8). The recording sites were scrubbed and cleansed to decrease electrical impedance. Electrodes were placed parallel to the muscle fibres, and a reference electrode was placed on the right lateral epicondyle of participants. A potentiometer attached to the central axis of each manipulandum was used to collect wrist angular position data. On each trial, raw band-passed (20-450Hz) EMG and unfiltered position data were digitally sampled for 3 s at 1 kHz using a 16 bit analog to digital converter (National Instruments Inc.) and stored for offline analysis. Data collection was completed using a customized LabVIEW program (National Instruments Inc.)

### Data reduction

Displacement onset was determined as the first point at which angular displacement of more than 0.2° occurred following the IS. If the incorrect wrist was used to respond, if a response was made in less than 100 ms or more than 500 ms following the go-signal, or if a response was made with both wrists in forced-choice trials, these responses were classified as “errors” and removed from analysis. In free-choice trials errors were noted only when a response was made with both wrists. This procedure resulted in the study-wide removal of 122/5760 of trials in the forced-choice conditions (2.1%), and 174/1440 trials in the free-choice conditions (12.1%).

EMG data were analyzed for differences in timing of burst onsets and offsets. Signals were rectified and filtered using a 25 Hz low pass elliptic filter, and displayed on a computer monitor using a customized LabVIEW program. Markers indicating EMG burst onsets in wrist extensors and flexors were then placed by the computer program on the EMG traces at the point in time at which EMG activity first reached a value 2 standard deviations above baseline levels (i.e., mean of 100 ms of EMG activity preceding the IS). Similarly, EMG offset markers were placed at the point in time when EMG activity first fell below 80% of peak activity and remained below for at least 25 ms. Activity between EMG onset and offset was defined as a distinct burst. EMG markers were manually adjusted (if necessary) to allow for correction of errors due to the strictness of the algorithm and these time points were recorded for analysis [16].

Kinematic variables included peak velocity, time to peak velocity, peak displacement, and time to peak displacement. Peak velocity was the maximum angular velocity achieved prior to reaching peak displacement and time to peak velocity was the time between movement onset and peak velocity. Peak displacement was the maximum angular displacement attained following movement onset, and time to peak displacement was the time between movement onset and this point.

### Statistical analysis

For forced-choice and free-choice trials kinematic variables as well as premotor RT (IS to EMG onset) were each analyzed using separate 3 (Polarity: anode right, anode left, sham) x 2 (Time: pre-tDCS, post-tDCS) x 2 (Response limb: left, right) Repeated Measures Analysis of Variance (RM ANOVA). Additionally, for free-choice trials the proportion of responses made with the right hand were analyzed using a 3 (Polarity) x 2 (Time) RM ANOVA. Prior to analysis, proportion variables were corrected for normality using an arcsine square root transformation. Post-hoc Student’s t-tests were administered where appropriate to determine the locus of any differences. Partial eta squared (η_p_^2^) is reported as an estimate of the proportion of the variance that can be attributed to the tested factor. Differences with a probability of less than .05 were considered significant.

## Results

### Forced-choice trials

**Fig 1:**
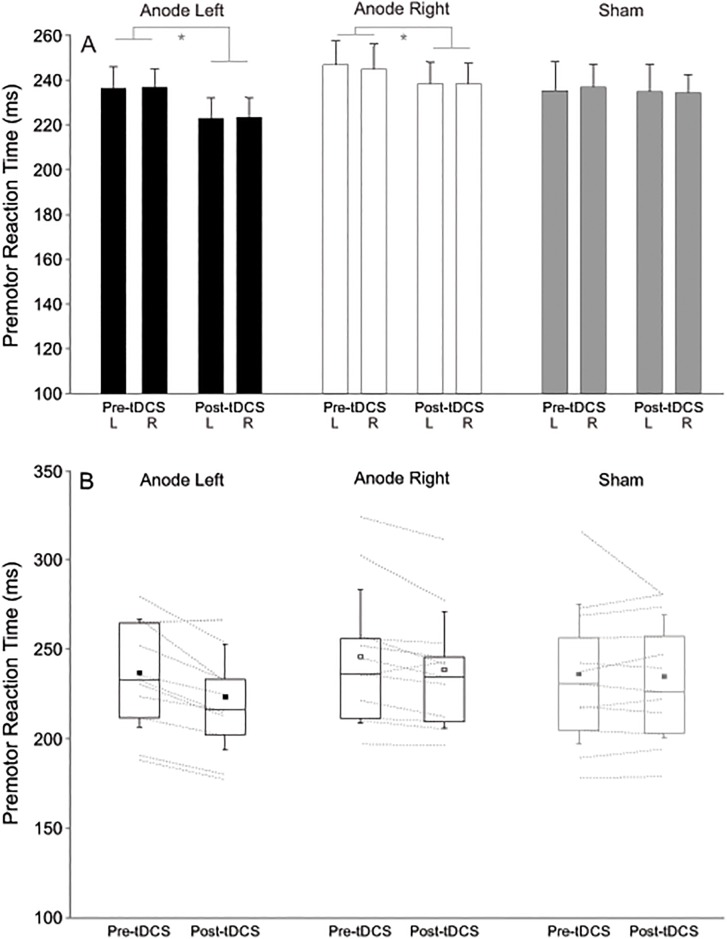
Mean premotor reaction time (ms) during forced-choice trials. A) Left (L) and right (R) hand reaction times are shown in pre- and post-tDCS blocks for each stimulation polarity (anode left, anode right, and sham). Error bars denote standard error, * indicates significant differece (*p* < .05). B) Boxplots of premotor reaction time in pre- and post-tDCS blocks (collapsed across response hand) where the small square indicates the mean, the horizontal line within the rectangular box indicates the median, and the boundaries of the rectangular box indicate the 25th and 75th percentiles. Dotted lines show individual participant data in each condition.

Premotor RT during forced-choice trials pre- and post-stimulation is presented in Fig 1. Analysis revealed a significant main effect of Time (pre- vs. post-tDCS), *F*(1,11) = 17.040, *p* = .002, η_p_^2^ = .963, but no main effect of Response limb, *F*(1,11) = 0.002, *p* = .969, η_p_^2^ = 0.001, or stimulation Polarity, *F*(2,22) = 1.295, *p* = .294, η_p_^2^ = .105. However a significant interaction between Polarity and Time superseded these effects, *F*(2,22) = 4.204, *p* = .028, η_p_^2^ = .277. Inspection of the data in Fig 1 indicates that premotor RT during forced-choice trials was significantly faster in both hands in the post- real tDCS blocks irrespective of stimulation Polarity, but not following sham stimulation. Post-hoc Student’s t-tests performed on premotor RT collapsed across hands between pre- and post-tDCS blocks confirmed that tDCS with the anode over the left motor cortex led to a significant, *t*(11) = 4.453, *p* = .001, *r* = .744, decrease in premotor RT (-13 ms, SD = 10), and tDCS applied with the anode over the right motor cortex led to a significant, *t*(11) = 3.091, *p* = .01, *r* = .611, decrease in premotor RT (-7 ms, SD = 8), whereas no difference (p = .696) was noted between pre- and post-tDCS blocks in the sham conditions (-1 ms, SD = 12).

Analysis of kinematic variables during forced-choice trials showed no significant main effects and no significant interactions between the variables (all *p*-values > .05), suggesting that a similar movement was produced across all conditions.

### Free-choice trials

**Fig 2:**
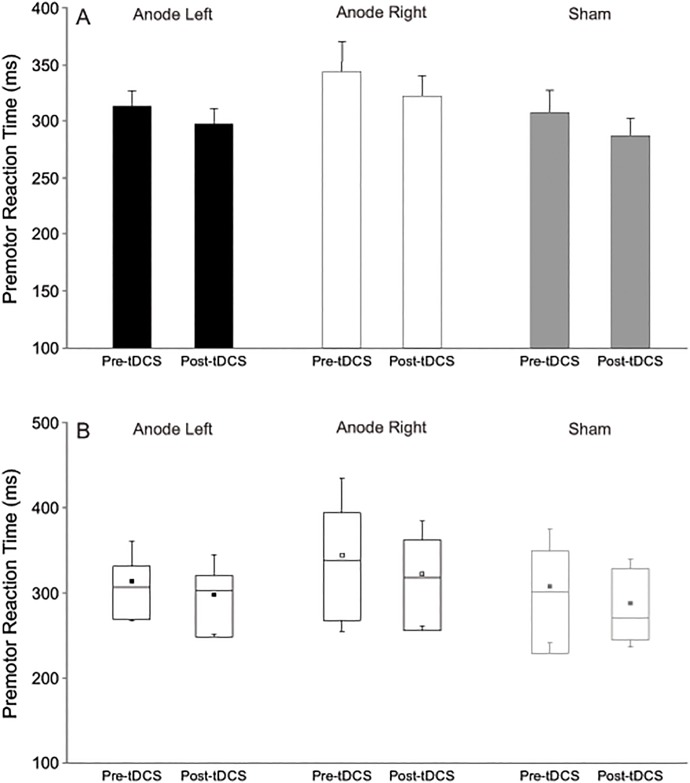
Mean premotor reaction time (ms) during free-choice trials. A) Reaction times collapsed across hand are shown in pre- and post-tDCS blocks for each stimulation polarity (anode left, anode right, and sham). Error bars denote standard error. Note that when collapsed across stimulation polarity there is a significant difference between pre- and post-tDCS blocks (*p* = .048). B) Boxplots of premotor reaction time in pre- and post-tDCS blocks for each stimulation polarity (collapsed across response hand) where the small square indicates the mean, the horizontal line within the rectangular box indicates the median, and the boundaries of the rectangular box indicate the 25th and 75th percentiles.

Analysis of the proportion of responses made with the right hand during free-choice trials revealed no significant main effects for Polarity, *F*(2,22) = 0.832, *p* = .448, η_p_^2^ = .07, or Time, *F*(1,11) = 1.099, *p* = .317, η_p_^2^ = 0.091, and no Polarity x Time interaction, *F*(2,22) = 0.315, *p* = .733, η_p_^2^ = .028. These results indicate that applying tDCS between blocks did not affect the response side chosen, with similar proportions of right hand responses pre-tDCS (59%, SD = 16) compared to post-tDCS (61%, SD = 18) regardless of which hemisphere received anodal stimulation. Analysis of premotor RT during free-choice trials (see Fig 2) revealed no main effect of Polarity, *F*(2,22) = 2.229, *p* = .131, η_p_^2^ = .168, or Response hand, *F*(1,11) = 2.635, *p* = .133, η_p_^2^ = .193, but there was a significant main effect of Time *F*(1,11) = 4.931, *p* = .048, η_p_^2^ = .310. No interaction effects were found between stimulation Polarity and Time (*p* = .961), Polarity and Response hand (*p* = .799) or Time and Response hand (*p* = .149). Finally the three way interaction effect between the factors was not significant (*p* = .094). These results indicate that in free-choice trials mean RT was slightly faster in the post-stimulation blocks (303 ms, SD = 37) compared to the pre-stimulation blocks (322 ms, SD = 54) irrespective of stimulation polarity/sham stimulation.

Analysis of kinematic variables during free-choice trials showed a significant main effect of Response hand for time to peak velocity, *F*(1,11) = 14.640, *p* = .003, η_p_^2^ = .571, time to peak displacement, *F*(1,11) = 6.887, *p* = .024, η_p_^2^ = .385, and magnitude of peak displacement, *F*(1,11) = 5.233, *p* = .043, η_p_^2^ = .322. There were no other significant main effects or interactions between the variables (all *p*-values > .05). These data indicate that in the post-stimulation blocks, peak velocity was reached sooner with the right hand (85 ms, SD = 19) compared to the left hand (75 ms, SD = 16), peak displacement was also reached sooner with the right hand (132 ms, SD = 30) compared to the left hand (142 ms, SD = 29), but that the magnitude of peak displacement was larger in the left hand (33.1 deg, SD = 5.9) compared to the right (29.5 deg, SD = 6.1).

## Discussion

The purpose of the current study was to determine whether the application of bi-hemispheric tDCS would lead to lateralized RT changes in a choice-RT task including both forced-choice and free-choice trials. Contrary to expectations, results clearly show that applying tDCS in a bi-hemispheric montage led to a decrease in RT for both right and left hand forced-choice responses (Fig 1), irrespective of whether the anode was placed over the left or the right motor cortex. Importantly, sham stimulation had no effect on forced-choice RTs which strongly suggests the effector- and polarity-independent effects are attributable to tDCS, rather than to a practice effect. In the free-choice trials, there were no differences in the proportion of responses made to each side and RTs were faster in all post-tDCS blocks including following sham stimulation (Fig 2), which indicates that applying tDCS bi-hemispherically had no effect on RT or the response side chosen.

There are several distinct possibilities related to the bi-hemispheric electrode montage that may explain the observed polarity-independent effects on RTs during the forced-choice trials. The first possibility is that bi-hemispheric tDCS did *not* actually cause an excitability increase in one motor cortex and related decrease in the other as hypothesised; however, this cannot be confirmed as excitability was not directly measured. Even though differences in cortical excitability have been shown previously following bi-hemispheric tDCS [17], they were relatively short lived (< 20 min) and induced using a lower intensity and shorter duration (2 mA for 5 min with a current density of 0.057 mA/ cm^2^) than that used in the current study. Interestingly, one study showed that propriospinal excitability was affected differently by bilateral tDCS (current density 0.056 & 0.029 mA/ cm^2^ for cathode & anode respectively [1 mA for 15 min]) compared to when tDCS was delivered unilaterally over the same location (0.029 mA/ cm^2^ [1 mA for 15 min]) [18]. The authors suggested that the differential effects were likely due to excitability changes in subcortical structures induced by bilateral tDCS compared to the previous cortical locus of excitability changes induced by unilateral tDCS [18]. In a similar way, the RT differences observed following bi-hemispheric tDCS in the present study (Fig 1) may have resulted from excitability changes in subcortical structures, as opposed to cortical changes. Based on recent computer modelling of tDCS current flow [19], a bi-hemispheric montage may actually result in diffuse current flow with the peak electric field occurring midway between the two electrodes rather than strong currents under each electrode. It is reasonable to suggest that bi-hemispheric tDCS may have had a larger effect on subcortical brain structures than on cortical ones. In fact, animal and human neuroimaging studies have revealed effects of tDCS on subcortical structures such as the reticulospinal tract [20], midbrain, and brain stem during bilateral tDCS [21]. It has been argued that many subcortical structures (e.g., the thalamus) are important contributors to the movement preparation and initiation circuits [22, 23] as they are located between motor areas of the cortex and other motor-related subcortical structures (e.g., basal ganglia & cerebellum). Thus, increased excitability in these regions may have led to the observed RT improvements.

A second possible explanation for the polarity-independent RT effects observed in the current study is that regardless of polarity, the bi-hemispheric tDCS led to increased activation under both electrodes. There is some recent evidence to suggest that cathodal stimulation delivered with a high current density results in cortical excitability enhancement instead of inhibition [24]. Specifically, Batsikadze and colleagues [24] found that cathodal stimulation delivered with a current density of 0.057 mA/ cm^2^ (20 min) actually increased cortical excitability, while cathodal stimulation delivered at 0.029 mA/ cm^2^ (20 min) resulted in the predicted decrease in excitability. Their study suggested that the previously described polarity-dependent directions of plasticity are no longer warranted when using higher tDCS intensities and/or longer stimulus durations (e.g., 2 mA tDCS for 20 min). When taking the surface area of the active electrode into consideration, this explanation suggests current densities above 0.057 mA/ cm^2^ may result in a reversal effect for cathodal stimulation. In the current study tDCS was delivered at 1 mA for 10 minutes; however, the small size of the active electrodes led to a current density of 0.128 mA /cm^2^ which is well above that reported by Batsikadze et al [24], suggesting that the high current density may well have led to increased excitability at both electrode sites. Similar to the polarity-independent performance enhancement found in the present study, a recent learning study which applied tDCS (2 mA for 25 min) bilaterally over the motor cortices with a current density of 0.057 mA/ cm^2^ found equivalent enhancements in finger sequence learning in the untrained hand which had received cathodal stimulation to that of the trained hand which received anodal stimulation [25].

A final possible explanation for the polarity-independent RT effects observed in the current study is that although previous research has shown that simple RT can be speeded or slowed using a unilateral tDCS montage [11], it is possible that a bi-hemispheric tDCS montage did not result in a similar behavioural effect, despite producing the expected change in neural excitability. Specifically, it is possible that, bi-hemispheric tDCS increased excitability in the hemisphere receiving anodal stimulation while it simultaneously decreased the excitability in the hemisphere underlying the cathode; as has been recently shown by Mordillo-Mateos and colleagues [17]. However, faster and slower RTs depending on the stimulation received by each cortex may not have been observed due to a concomitant change in interhemispheric dynamics. For example, Sehm and colleagues [21] have demonstrated that the effects of unilateral and bi-hemispheric tDCS over M1 are different, and that these effects are network-wide, and can change following the end of stimulation compared to during stimulation. Specifically, they found that while no differences were observed between montages during stimulation, once stimulation had finished bi-hemispheric tDCS induced bilateral but opposing changes in intracortical functional connectivity (i.e., a decrease in functional connectivity ipsilateral to the side receiving anodal stimulation along with a contralateral increase in connectivity) [21]. As such, in the current study, the unexpected observed speeding of forced-choice RT contralateral to the cathodal stimulation may be explained by beneficial intracortical changes driven by the cathodal stimulation over the opposite motor cortex. These functional changes might be explained by the direction of the current flow as it passed through the brain. Bi-hemispheric tDCS induces current directed side to side (i.e., along the coronal plane) in the brain, whereas a unilateral tDCS montage induces current front to back or vice-versa (i.e., along the sagittal plane) [18], which may be important for determining the direction and degree of neural excitability changes [26]. To date, the limited evidence suggests that this observed polarity-independent effect of bi-hemispheric tDCS is a result of dynamic interhemispheric and intracortical changes specific to the bilateral montage [21, 27]. In addition, functional benefits of bi-hemispheric tDCS (as opposed to unilateral tDCS) have recently been used in neurorehabilitation therapy to enhance functional motor recovery in stroke patients [28]. The enhancement of motor recovery are thought to occur as a result of the dynamic interhemispheric interactions caused by the bilateral stimulation montage [27], which may similarly account for the bilateral polarity-independent improvements in forced-choice RTs shown in the present experiment.

In the free-choice conditions there appeared to be no effect of tDCS on the behavioural outcome. Specifically, there was no change in the proportion of responses made with the right hand following either real or sham stimulation. Yet, in the free-choice trials a significant reduction in RT was observed between the pre-tDCS and post-tDCS blocks for all stimulation sessions, including the sham session (Fig 2). Because a similar reduction in RT was seen following sham stimulation this result cannot be attributed to the tDCS and is more likely to be the result of practice effects. Because no practice effects were seen in the forced-choice trials as evidenced by a lack of pre-post tDCS RT differences during the sham session (Fig 1A), it may be speculated that the free-choice condition involves a much more complex and cognitive mode of control which is less influenced by cortical and/or subcortical motor excitability changes. Indeed, it is not uncommon to observe RT improvements in more complex tasks over a short period as observed here [29]. The kinematic analyses also suggest that the free-choice condition may have been more complex as there was a tendency towards slower (time to peak velocity and time to peak displacement) and less accurate movements with the left (non-dominant) limb. These differences, however, were absent during the forced-choice condition.

Although the precise mechanism and locus of neural excitability changes accompanying bi-hemispheric tDCS are unclear, the present study shows evidence of an effector-independent speeding of response initiation in a forced-choice RT task following bi-hemispheric tDCS delivered at 1 mA for 10 min with a current density of 0.128 mA/ cm^2^. It is self-evident, given the multiple explanations outlined above, that further investigation into the physiological effects of tDCS (including but not limited to the impact of stimulation parameters and electrode montages) is warranted. Moreover, inconsistencies in the amount of current applied (mA), duration of stimulation (minutes), and current density (mA / cm^2^) between tDCS studies adds to the difficulty in drawing firm conclusions. Here, the present work yields novel information regarding the functional effect of bi-hemispheric tDCS on motor processes, indicating that irrespective of current flow direction, forced-choice RT is facilitated following tDCS applied bi-laterally over the motor cortices.
